# The Role of Yeasts in Human Health: A Review

**DOI:** 10.3390/life13040924

**Published:** 2023-03-31

**Authors:** Cátia Filipa Caetano, Carlos Gaspar, José Martinez-de-Oliveira, Ana Palmeira-de-Oliveira, Joana Rolo

**Affiliations:** 1 CICS-UBI—Health Sciences Research Center, Faculty of Health Sciences, University of Beira Interior, 6200-506 Covilhã, Portugal; catia.caetano@ubi.pt (C.F.C.);; 2 Faculty of Health Sciences, University of Beira Interior, 6200-506 Covilhã, Portugal; 3 Labfit-HPRD: Health Products Research and Development Lda, 6200-284 Covilhã, Portugal

**Keywords:** *Candida*, *Cryptococcus*, *Malassezia*, *Rhodotorula*, mycobiome

## Abstract

The microbiome consists mostly of bacteria, but new evidence and developments in sequencing methods have shown that fungi play an important role in human health and in the stability of the microbiota. Scientific knowledge about the role of commensal fungi in intestinal, oral, vaginal and cutaneous communities has been increasing; however, more studies are still needed to better understand their action in these niches. To date, fungal research focuses primarily on opportunistic diseases caused by fungal species, leaving unclear the possible role of fungi as an integral part of the microbiota. Although they are much less abundant than bacteria, fungi such as species belonging to the genus *Candida*, *Malassezia*, *Rhodotorula* and *Cryptococcus* are some of the yeasts that have been in the focus of the scientific community because they inhabit various niches. In this review, we have summarized the current information about the yeasts that inhabit the human body, including some of the diseases that they can cause when the microbiota becomes unstable.

## 1. Introduction

**Figure 1:**
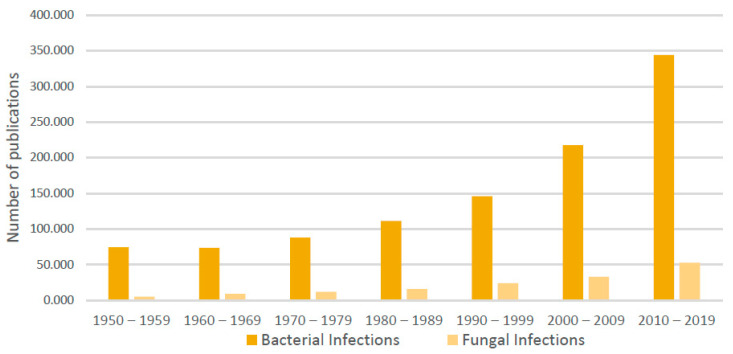
Number of publications on PubMed regarding bacterial infections (dark yellow) and fungal infections (light yellow) per decade, on the last 60 years.

The set of microorganisms that usually live inside the human body, also called human microbiota, generates the microflora. Its genomic constitution and its by-products are called the human microbiome [1]. This microbiome is unique for each individual and is never stable, due to environmental, nutritional and ecological changes. Furthermore, in everyone, due to environmental variations, each body site is home to a distinct microbial ecosystem [2]. Microorganisms can adapt to different survival conditions. *Bacteroidetes* and *Firmicutes*, followed by *Proteobacteria*, *Fusobacteria*, *Tenericutes*, *Actinobacteria* and *Verrucomicrobia* were reported to be the most dominant, constituting about 90% of the total microbial population in humans [3]. A substantial amount of information is readily available regarding the human microbiome; much less is known regarding the human mycobiome, i.e., the type and number of fungal species that inhabit the human ecological niches. In fact, studies focusing on yeast infections are scarce compared with studies focusing on bacterial infections (Figure 1).

Several human ecological niches are colonized by millions of different microorganisms, of which the gut is by far the most studied. The gut is recognized as a virtual organ closely associated with the health and longevity of the host. The gut microbiome has both beneficial and adverse impacts on gut tissue homeostasis [4]. From a very early stage of life (after 3 years of age), the microbial composition of the intestinal microbiome varies over time, never managing to remain 100% stable [4]. The intestinal mycobiome is constituted in abundance by fungi, such as *Candida* spp., *Aspergillus* spp., *Fusarium* spp. and *Cryptococcus* spp., which can have a pathogenic effect on the host [5].

Although most oral microbiome studies have been performed on bacteria, there has been a recent surge in studies on oral fungal communities [6]. Over the past few years, exploration of the composition of the mycobiome in oral samples has shown that they were colonized by *Candida* spp., *Cladosporium* spp., *Saccharomyces* spp., *Penicillium* spp., *Malassezia* spp., *Aspergillus* spp., *Cryptococcus* spp., *Rhodotorula* spp. and *Trichosporon* spp., among others [6].

Characterization of fungal communities over time has revealed that mode of delivery influences colonization by *Candida* species, where the relative abundance of *Candida albicans* is highest on the skin of vaginally delivered infants [7]. On the other hand, characterization of the fungal communities in the skin has revealed abundant colonization by *Malassezia* spp., *Candida* spp., *Cladosporium* spp., *Fusarium* spp. and *Cryptococcus* spp. [8]. In the vagina, although much less abundant than bacteria (values less than 1%), fungi, namely *Candida albicans*, have been identified as the most predominant microorganism affecting vaginal health. One study demonstrated the presence of *Candida*, *Clavispora lusitanie*, *Malassezia*, *Rhodotorula*, *Aspergillus* and *Leptosphaerulina* as some of the most prevalent fungi in the vaginal mucosa [9].

With this review, we intend to obtain knowledge about the composition and role of commensal fungi in human health, with special focus on fungal microorganisms that can colonize the skin, vagina and mucous membranes.

## 2. Materials and Methods

This review was based on a literature search to obtain a blend of different types of systematic reviews, as well as to obtain information about yeasts in different niches (focusing mainly on the intestinal, vaginal and oral mucosae). The articles and dissertations mentioned were obtained through the following platforms: Scielo; PubMed and Web of Science. The titles of the subjects and the keywords chosen were mycobiome, intestinal microbiota, skin yeasts, fungi, vaginal microbiota, oral microbiota, vulvovaginal diseases and skin diseases. The inclusion criteria were language (Portuguese and English), text availability and preferred publication date (last 5 years).

## 3. Results

### 3.1. Frequent Colonizers of Human Ecological Niches

The different mycobiomes in the human body not only influence a particular niche, but rather, a range of niches in the human body, due to the interrelationships between the different microbial communities.

In recent years, there has been an increase in human invasive fungal infections, mostly caused by pathogens such as *Candida* spp.; however, some less common ones, such as *Rhodotorula* spp., have been recognized as emerging pathogens [10]. Transmission of mycobioma of the skin and gastrointestinal tract is thought to be via the vaginal route during delivery, composed mostly of Candida albicans [11].

Although the skin is the niche where *Malassezia* is most commonly found, in recent years it has been identified in other unrelated niches, including the human gut, breast milk and internal organs, including those of the central nervous system [10]. Transmission of *Malassezia* spp. by this way is suggested, although there is controversy as to whether transmission is initiated from the placenta [12]. Therefore, the fungal diversity of the skin and gut of newborn babies is derived from the maternal mycobioma [13]. 

**Figure 2:**
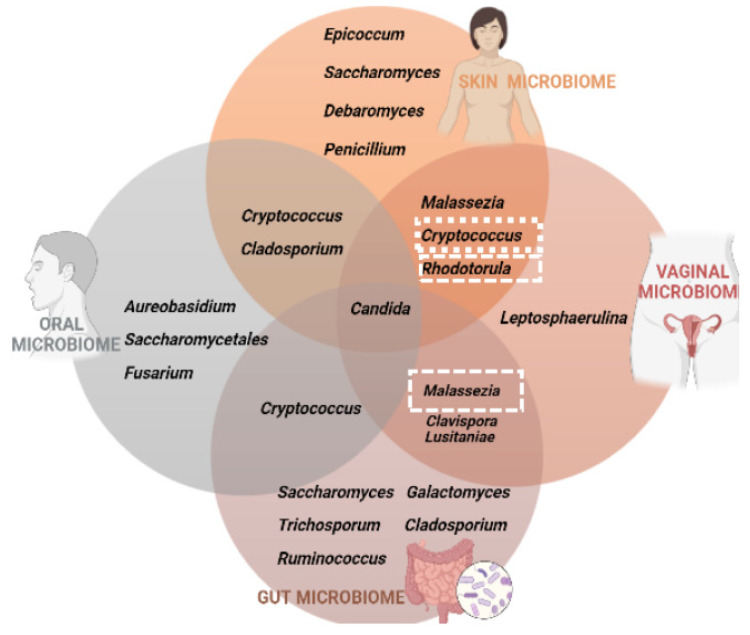
Summary of the various fungal communities that influence the stability of the skin, vagina, gut and oral microbiota. In the dashed box we emphasize the yeasts that will be discussed throughout this article.

*Cryptococcus neoformans* is a known human pathogen, particularly in the immunocompromised host. However, other species of this genus can be also found colonizing several human niches [14]. In Figure 2, we summarize the fungal communities that inhabit the four different niches under analysis. We were able to conclude that *Candida* spp. is the common fungus present in the microbiota of the skin, vagina, intestine and oral cavity; on the other hand, the three other yeasts analyzed in this review, that correspond to the ones that are frequent colonizers of the human body but also are human pathogens, do not inhabit a niche only. Therefore, our research in this subject has revealed that these four genera possibly comprise opportunistic yeast species.

#### 3.1.1. *Candida* spp.

This genus is one of the most commonly found in the normal human microbiota, managing to colonize niches such as oral mucous membranes, skin, gastrointestinal, genital and urinary tracts without causing infections. However, in immunocompromised people or people with chronic illnesses, this yeast can become pathogenic, thus causing infections called candidiasis [15]. Within this genus, there are species that are one of the most frequent causes of opportunistic infections, *Candida albicans*, the drug resistant, *C. glabrata*, the new global threat to public health, *C. auris* and other emerging species such as *C. tropicalis*, *C. parapsilosis* and *C. krusei* [16,17]. *C. albicans* is a yeast that can reside in the human body while also living in certain environmental reservoirs [18]. Disturbances induced either by antibiotics, immune system anomalies, alterations in the microbiome and/or alterations in the integrity of the mucocutaneous barrier allow *Candida* spp. to become an opportunistic pathogen in the context of a series of virulence factors [19,20].

*C. albicans* colonization has been found in the vaginal niche by culture-dependent methods in approximately 20% to 30% of asymptomatic women and by molecular methods in about 65% [21]. When the infection is symptomatic, it is defined as vulvovaginal candidosis (or candidosis), 80% to 92% of which are caused by *C. albicans* [22].

The success in the occurrence of infections comes from specific characteristics of this genus. One is the change from a yeast form to a branched hyphal form. As a rule, the yeast form is associated with commensalism, dissemination, or transmission of the infection [23]. The balance between the two forms is maintained by the responses of the innate immune system [24].

#### 3.1.2. *Cryptococcus* spp.

The genus *Cryptococcus* is a group of capsulated opportunistic fungi. Of the 37 species discovered, *C. neoformans* and *C. gattii* are the species that most cause pathogenesis in man [14]. *Cryptococcus* spp. is an environmental yeast and is therefore very recurrently found in nature (soil, animal feces, among others). *C. neoformans* essentially affects immunocompromised individuals (elderly people, patients with HIV/AIDS and recently transplanted organs) in addition to causing infections of the central nervous system [25]. It is estimated that annually, 223,000 people with HIV/AIDS develop cryptococcal meningitis, of which 181,000 eventually die [26].

*C. gatti* manages to infect both immunocompetent and immunosuppressed individuals, obtaining a mortality rate of almost 33% [27]. That said, this genus has become a lethal pathogen that poses a serious threat to public health [27].

#### 3.1.3. *Malassezia* spp.

This yeast is included in the genus Basidiomycota, class *Malasseziomycetes* and family *Malasseziaceae* [28]. *Malassezia*, a commensal yeast that is lipophilic and lipid dependent for its growth, except *M. pachydermatis*, is the main component of the fungal skin microbiota of many mammals, corresponding to more than 90% of the total fungal population in the skin niche [29].

The mechanisms by which these yeasts can trigger diseases are poorly studied and, therefore, are not yet clearly identified. However, the proposed hypothesis is that the diseases can be induced by direct invasion of fungal filament tissue or indirectly by immunological and metabolic mechanisms [30,31,32,33].

Studies already carried out on the composition of the fungal microbiota of breast milk revealed two new pieces of information [32,34]: in its composition, the proportion of fungi is much greater than that of bacteria (contrary to what is found in many other niches) and among the fungi found, 40% of the total of genera identified by pyrosequencing are represented by *Malassezia*.

Different species of *Malassezia* spp. can lead to different clinical manifestations, which can range from hypopigmentation without visible inflammation, to eczema with scaling and inflammation. Of all the skin diseases, although still controversial, malassezia is a pathogenic agent of pityriasis versicolor and malassezia folliculitis. The discovery of this yeast in seborrheic areas still needs further study since its commensal state is still difficult to distinguish from its pathogenic phase [35]. Scientific evidence for the role of *Malassezia* in psoriasis and atopic dermatitis is less robust than for other diseases [36].

#### 3.1.4. *Rhodotorula* spp.

The pathogenic role and its relevance in the intestinal microbiota have not been studied nor seen as important by researchers, although they are frequently found in human stool samples [37,38]. However, human fungal infections by *Rhodotorula* spp. have been increasing in recent years [39]. Until 2018, clinical cases have been reported, where of the 40 species, *R. mucilaginosa* is the most common cause of human infection (72%), followed by *R. glutinis* (21%) and *R. minuta* (7%) [40,41,42].

This yeast can be found as contaminants of the skin, nails, lungs, urine, feces, central nervous system and blood. The infection can be installed endogenously or exogenously, as well as by the hands of health professionals, contaminated materials and inanimate environmental sources [43]. In addition to these transmission routes, *Rhodotorula* spp., being an environmental yeast, can be found in nature by isolating it from environmental sources such as air, soil and plants [44].

### 3.2. Mycobiomes in Various Body Niches and Their Associated Diseases

It is estimated that there are about three million species of fungi, of which about 300 cause infections in humans (Table 1) [45]. In studies previously performed, the authors identified about 66 different genres of fungi present, the most prevalent being *Candida*, *Cladosporium* and *Saccharomyces* [46]. 

The ecological relationships between bacteria, archaea, viruses and fungi maintain the host’s equilibrium and are part of several vital processes, such as nutrition and protection against pathogens. Being one of the largest eukaryotic kingdoms, fungi have a variety of life cycles with adaptations in metabolism and morphogenesis that allow them to adapt to environmental changes and give them survival throughout the human body, such as the intestine, skin, oral cavity, urogenital tract and digestive system [47]. There is a correlation between the occurrence of changes in the fungal community and the diseases caused in humans.

**Table 1:** Illnesses that are caused by colonizing fungi. The upward and downward arrows represent the increase and decrease, respectively, of fungi colonisation in the various diseases.

Human Ecological Niches	Diseases	Yeast Colonization	References
Gut	Inflammatory bowel disease (Crohn’s disease)	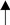 *C. albicans*, *C. tropicalis*, *C. glabrata*, *Aspergillus clavatus*, *C. neoformans*, *Cyberlindnera jadinii*, *Clavispora lusitaniae*, *Debaryomyces hansenii*, *Kluyveromyces marxianus* 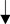 *Saccharomyces cerevisiae*	[48,49,50,51]
	Irritable bowel syndromes (IBS)	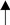 *Candida* spp., *Malassezia* spp., *Cladosporium* spp., *Saccharomyces cerevisiae* 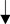 *Mycosphaerella* spp., *Aspergillus* spp., *Sporidiobolus* spp., *Pandora* spp.	[52,53]
	Colorectal cancer	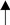 *Trichosporon* spp., *Malassezia* spp.,	[54]
	Obesity	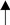 *Candida*, *Nakaseomyces*, *Penicillium*, *Porphyromonas*, *Campylobacter*, *Bacteroides*, *Staphylococcus*, *Parabacteroides*, *Dialister* and *Ruminococcus* 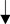 *Mucor racemosus* and *M. fuscus*	[55,56]
	Diabetes	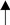 *C. albicans and Saccharomyces* spp. In type 1 Diabetes 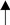 *C. albicans*, *Cladosporium* spp., *Meyerozyma* spp., *Mortierella* spp. and *Aspergillus* spp. In type 2 diabetes	[55,57,58]
Skin	Pityriasis versicolor	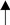 *M. globosa and M. sympodialis and M. furfur.*	[59]
	Seborrheic dermatitis	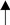 *Malassezia* spp.	[60]
	Psoriasis	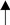 *M.biota; M.sympodialis; Kocuria*, *Lactobacillus* and *Streptococcus* with *Saccharomyces*,	[61,62]
Oral	Oropharyngeal candidosis	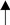 *C.albicans*	[63]
	Dental cavity	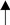 *C.albicans*, *C. dubliniensis*, *Debaromyces* spp., *Rhodotorula* spp., or *malassezia* spp.	[64,65,66]
	Periodontitis	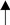 *C. albicans* 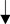 *S. cerevisiae*	[67,68]
Urogenital	Vulvovaginal candidosis (VVC)	* 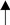 * *C. albicans*	[8]
	Candiduria	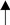 *C. albicans*, *C. glabrata* and *C. tropicalis*	

#### 3.2.1. Gut Mycobiome

The most studied fungal phyla present in the human intestine have been *Ascomycota* as the most predominant phylum in the intestine, followed by *Zygomycota* and *Basidiomycota* [69,70,71]. A newborn’s gut microbiome is highly dependent on its diet. Breastfed babies have higher levels of bacteria belonging to the Actinobacteria class [72,73] and the genera belonging to *Lactibacillus* and *bifidobacterium* [72,73,74,75]. High amounts of oligosaccharides and various fatty acids that make up breast milk positively influence the intestinal microbiome and their metabolites may help to act against hypersensitivity (allergy) and asthma counter reactions [76].

*Saccharomyces* is a non-pathogenic yeast, thermotolerant and resistant to the action of gastric, enteric and pancreatic juices. *Saccharomyces* has been used commercially in the production of probiotic foods. In recent decades, *S. cerevisiae* and *S. boulardii* have shown great promise as probiotic treatments [77]. Several studies have shown *S. cerevisiae* and *S. boulardii* to be associated with an increase in the proportion of *Bacteroidetes* in the gut microbiota composition and a decreased relative abundance of *Firmicutes* and *Proteobacteria*. Furthermore, this yeast can prevent inflammation by promoting pro-inflammatory immune function and increasing the production of short-chain fatty acids [77,78,79,80].

Other studies have also reported that *Malassezia*, *Candida* and *Saccharomyces* constitute the intestinal microbiota, with *S. cerevisiae*, *M. restricted* and *C. albicans* identified in 96.8%, 88.3% and 60.8% respectively [81]. Similar to other microbial communities, fungi also have a high capacity to produce metabolites that can be applied for medicinal or therapeutic purposes. In addition to this functionality, they can also influence host homeostasis, causing biological effects on them as part of fungus–host interactions [82].

##### Inflammatory Bowel Disease

Changes in the gut microbiome are associated with increased disease and dysbiosis of fungal communities which may contribute to susceptibility or increased disease severity. It is important to note that these changes, when caused by external influences, will facilitate invasion and that gastrointestinal (GI) infections affect the composition of the microbiota.

In CD (Crohn’s disease), there is an increase in the fecal fungal burden as well as an increase in the *Basidiomycota*—*Ascomycota* ratio. Regarding the opportunistic yeasts that cause this disease, an increase in the abundance of *C. albicans*, *C. tropicalis*, *C. glabrata*, *Aspergillus clavatus*, *Cryptococcus neoformans*, *Cyberlindnera jadinii*, *Clavispora lusitaniae*, *Debaryomyces hansenii* and *Kluyveromyces marxianus* and a decrease in *Saccharomyces cerevisiae* have been found [48,49,50,51].

##### Irritable Bowel Syndromes (IBS)

Although not considered a critical illness, it affects about 10–15% of individuals, reducing their quality of life [30]. Studies have found an increase in the prevalence of *Candida* spp. (notably *C. albicans*), *Malassezia* spp., *Cladosporium* spp. and *Saccharomyces cerevisiae*. In contrast, yeasts such as *Mycosphaerella* spp., *Aspergillus* spp., *Sporidiobolus* spp. and *Pandora* spp. suffer a decrease in their abundance [52,53,83,84].

##### Cancer

The intestinal microbiome and its changes have been associated with the pathogenesis of diseases such as colorectal adenoma, which induces colorectal cancer (CRC), esophageal squamous cell carcinoma (ESCC), gastric cancer, hepatocellular carcinoma (HCC), cholangiocarcinoma and pancreatic ductal adenocarcinoma (PDAC) [85].

Patients with these pathologies and with polyps have been observed with fungal dysbiosis, having a very high *Ascomycota/Basidiomycota* ratio leading to the expansion of opportunistic fungi [55].

Species such as *Trichosporon* and *Malassezia* were considered two of the populations capable of facilitating the progression and growth of colorectal cancer [54]. Other studies have found that although no single type has been identified as the single leading cause, there is evidence to show an association between *Fusobacterium* species (*F. mortiferum*, *F. nucleatum*, and *F. necrophorum*) with colorectal cancer [37]. Lev et al. also demonstrated that people with this pathology contain higher percentages of *Klebsiella*, *E. coli*, *Streptococcus* and *Enterococcus* as opposed to *Rothia* which is in low amounts [86].

##### Obesity

There are many causes that can lead to obesity, such as genetic, lifestyle and environmental factors. However, in addition to these causes, the intestinal microbiota plays a key role in the presence and development of obesity. The microbiota of overweight individuals has a greater capacity for fermentative processes and for capturing energy from the diet. It also has a high proportion of *Firmicutes* to *bacteroides*/*Prevotella* leading to an increase in the microbiota gene involved in polysaccharide degradation and an increase in SCFAs [87].

*Candida*, *Nakaseomyces*, *Penicillium*, *Porphyromonas*, *Campylobacter*, *Bacteroides*, *Staphylococcus*, *Parabacteroides*, *Dialister* and *Ruminococcus* have been the most identified genera in overweight individuals while Mucor racemosus and M. fuscus have been found the most in non-obese patients [55,56]. It is now obvious that obesity is somehow associated with gut dysbiosis, low-grade inflammation and a host of metabolic disorders.

##### Diabetes

Autoimmune diseases, unlike IBD, are directly linked to an abnormal development of the intestinal microbiota throughout life. A study on the quantitative changes in *Candida* species in patients with real-time PCR (qPCR) DM1 and DM2 was carried out, where *C. albicans* was the most common strain found in the stool of these patients [55]. However, no significant changes were observed between DM1 and DM2 patients in terms of *C. albicans* colonization [55].

Regarding genetics, nutrition and lifestyle choices, these also influence the prevalence of DM1, particularly if we are to assess the incidence rate in various countries around the world. Other studies have reported, in addition to an increase in *C. albicans*, that in type 1 diabetes there is also an increase in the genus *Saccharomyces* [57]. In type 2 diabetes, in addition to an increase in *C. albicans*, there an increase in *Cladosporium* spp., *Meyerozyma* spp., *Mortierella* spp. and *Aspergillus* spp. has also been found [58].

In addition, it has been speculated that the high prevalence of *Saccharomyces* may be due to the consumption of foods containing yeast (beer and bread), while the high percentage of *Candida* is related to the consumption of carbohydrates [46].

#### 3.2.2. Oral Mycobiome

Despite the abundance of the mycobiome, the candida genus remains one of the few that unquestionably contributes to the emergence of the most common infections in the oral mucosa [6]. However, although fungi that are present in a smaller percentage do not have a significant influence on metabolic activities, they can play a modulator role in immune responses or an opportunistic pathogenic role under surveillance conditions, harming the host [6]. In some rare cases, involving severe immunosuppression, *Cryptococcus* spp. and *Aspergillus* spp. were described as causing lesions in the oral mucosa [58].

##### Oropharyngeal Candidosis (OPC)

OPC can be classified into three main conditions: acute, chronic and chronic mucocutaneous candidosis [63]. Some of the risk factors include nutritional deficiencies, local dysbiosis, salivary hypofunctions, smoking, use of dentures and a dysfunction in T-cell immunity [63]. *C. albicans* is the fungus responsible for causing this disease, and life-threatening systemic infections can develop when this fungus enters the bloodstream [63].

##### Dental Cavity

The role of the oral mycobiome in caries has been a recent focus. A study by Baraniya in 2020 found that advanced caries were associated with an abnormal increase in the prevalence and abundance of *C. albicans* in adults and of *C. dubliniensis* in children [64,65].

Furthermore, one study reported a regressive trend in mycobiome diversity as caries severity increased [66]. Interestingly, in another study, *C. albicans* was associated with severe disease, while *C. dubliniensis* was shown to have a gradual and steady increase as the disease set in and grew [66]. On the other hand, in caries-free children, one of the most common fungi was found to be *Malassezia globosa* [64].

In summary, it is thought that *C. albicans* will be involved in more advanced lesions while *C. dubliniensis* plays a pivotal role earlier in the disease process. Some investigations suggest that some fungi are found in caries-free children, namely *Debaromyces* spp., *Rhodotorula* spp. or *malassezia* spp. [64,66].

##### Periodontitis

Although fungal communities have already been detected in the subgingival plaque, their role is still unclear. Research has reported an increase in yeast detection, namely *Candida* spp., in subjects with periodontitis [67]. However, this study was carried out through cultures, because when tests were carried out at the molecular level, this increase was not verified [68]. This study saw that this pathogenesis was associated with a decrease in microbiome diversity and changes in the relative richness of two genera (decrease in *S. cerevisiae* and an increase in *Filobasidiales* species) when compared to individuals with or without mild disease [68].

#### 3.2.3. Skin Mycobiome

Most studies on the microbiome focus on understanding the bacterial composition, but the microorganisms present on the skin are not limited to bacteria, but also include viruses, fungi and arthropods. Notably, the skin being the largest organ in the human body, it serves as a defensive obstruction against possible injury and microbial attack [36].

##### Pityriasis Versicolor

Pityriasis versicolor is a chronic skin infection characterized by discrete or confluent, scaly, dark or depigmented patches, mainly on the upper torso, but which can extend to the neck, abdomen and other locations. The *Malassezia* species identified mainly in pityriasis versicolor lesions are *M. globosa* and *M. sympodialis* and *M. furfur*. In addition to the findings of the action of this yeast in pityriasis versicolor, there are two facts that further emphasize its role in this disease: (i) a positive culture is found more in samples cultured from skin lesions than from healthy skin [59], and (ii) in its hyphal form, it is usually found in samples obtained from pityriasis versicolor lesions, regardless of the species of malassezia present [88].

##### Seborrheic Dermatitis

The relationship between malassezia colonization and seborrheic dermatitis was first described by Louis-Charles Malassez in 1874. Seborrheic dermatitis is an inflammatory dermatosis with a predilection for anatomical areas with a high concentration of sebaceous glands, such as the middle third of the face, chest, back and scalp. The prevalence of seborrheic dermatitis also increases with age (2.0% in <35 years; 3.6% in 35–64 years; 4.4% ≥65 years) and there is an association with other fungal diseases such as tinea pedis, onychomycosis and pityriasis versicolor. The age dependence of seborrheic dermatitis is probably responsible for the increased prevalence (14.3%) reported in the Rotterdam study [89].

Fungi such as *Malassezia* is found in the sebaceous gland where lipids are the main source of energy. As this yeast is usually commensal, the mechanism that triggers this disease has been increasingly investigated [60]. Its DNA has been detected in about 90% of skin lesions; the skin lesions of atopic dermatitis and colonization increase with the severity of the disease [90].

##### Psoriasis

Psoriasis is an inflammatory skin disease that affects approximately 2% of the world’s population. Little is known about the role of the skin microbiome in psoriasis. However, some studies have already been carried out on this disease. In one study, an increase in *Brevibacterium*, *Kocuria palustris* and *Gordonia* was found to be associated with back and elbow injuries [61]. In that same study, it was also found that there was a high abundance of the *Malassezia* fungus. Strains such as *M. biota* were detected on the coast, while *M. sympodialis* dominated elbow mycosis [61].

In psoriatic elbow skin, there was found to be a significant correlation between the occurrence of *Kocuria*, *Lactobacillus* and *Streptococcus* with *Saccharomyces*, which was not observed in healthy skin [62].

#### 3.2.4. Urogenital Mycobiome

The composition of the female microbiota varies according to age, hormone production, menstrual cycle, drug use and sexual activity. Evidence has shown that the composition of the microbiota is important for reproductive and genital tract health.

##### Vulvovaginal Candidosis (VVC)

Mucosal infections, characterized by fungal colonization associated with an inflammatory host response, are extremely common and cause a negative impact on the quality of life of patients.

VVC is a multifactorial condition with an abnormal immune system response. Undeniably, colonization is carried out by both bacteria and fungi, with communication between them through physical interactions, production of metabolites and chemical interactions, among others. Most in vivo studies have revealed an inhibitory capacity on the part of *Lactobacillus* on the growth, dimorphic transition, virulence factors and biofilm formation of *C. albicans* [8]. For example, about 75% of women of childbearing age have at least one episode of VVC and up to 9% of them suffer from recurrent VVC (more than four episodes per year) [63,91,92]. Some of the risk factors associated with this pathogenesis include a very high level of estrogen, the use of oral contraceptives and uncontrolled diabetes [63].

##### Candiduria

Most clinicians accept that Candiduria is defined as colonization or contamination, but it may be the only sign of invasive candidosis. The consensus is that Candiduria is quite common in hospitalized patients [93,94]. The spread of the disease usually happens through contamination. It is usually diagnosed in elderly patients, with *Candida* being the most frequently isolated microorganism in nosocomial urinary tract infections (UTIs). Overall, *C. albicans* accounts for 50 to 70% of cases, followed by *C. glabrata* and *C. tropicalis*, which is the third most common species. There are studies that claim that the use of antibiotics is associated with an increase in their incidence; however, these estimated values can be disregarded, as the standard urine culture is not very sensitive [95].

From all the studies carried out in patients with Candiduria, a set of risk factors that are associated with an increase in its incidence was gathered. In diabetes mellitus, frequent or prolonged use of antimicrobials, broad-spectrum antibiotics (which suppress the gastrointestinal and genital flora), can lead to ICU stay and use of indwelling catheters, among others.

### 3.3. Interconnection between Different Niches

Studies indicate that alterations in intestinal microbial diversity (dysbiosis) can lead to an increase in host susceptibility and an interruption of mucosal immune tolerance, which will influence skin health in the future [96]. Other studies have associated a bidirectional interaction between gastrointestinal health and skin homeostasis through the metabolic activity and immune impact of the gut microbiome [97,98]. For example, commensal intestinal microorganisms can promote skin allostasis by controlling T-cell differentiation [97].

Although a healthy gut microbiota is critical to host health, overgrowth of the host and changes in diversity can also result in disease processes, for example, patients with Crohn’s disease are also found primarily with psoriasis as a comorbidity [99,100].

Studies carried out deepening the knowledge of the possible relationship between the intestinal and oral microbiome reported that patients with intestinal diseases exhibited a considerable abnormal increase in oral microorganisms in the lumen and in the tissues of the intestinal mucosa [101,102]. Thus, it is plausible to believe that the cavity serves as a reservoir; however, we have still not obtained comprehensive information on which microorganisms act as pathogens.

Some researchers have already found evidence that suggests the existence of a gut–vagina axis. There are already some data that the intestinal and vaginal microbiota can be completely linked, for example, in the onset and progression of endometriosis [103]. Yet, another study about this connection between endometriosis and the intestine showed that after 42 days of persistence of endometrial injury, a distinct intestinal microbiota develops [104], that is, not only does the intestinal microbiota change the vaginal one, but vice versa.

These hypotheses open many doors to new preventive, diagnostic and therapeutic possibilities, and are therefore an emerging area for investigation.

## 4. Conclusions

With this review, we intended to synthesize the existing literature about the microbiota and mycobiota. In addition, we highlighted the communities that live in the different niches of the human body, namely the oral, vaginal, intestinal and skin cavities, as well as the diseases that occur when there are changes in the composition of the microbiota.

It should be noted that the importance and significant role that fungal communities play in human health are becoming increasingly more evident. However, more studies are needed to understand the effect of geography on the human mycobiome. Other aspects such as dietary habits or age should also be studied in detail to provide a deeper knowledge on the dynamics of the human mycobiome.

Furthermore, future research involving the study of the mycobiome should have the evolution and improvement in the health of humans, animals and also plants as an objective, as well as the improvement in the ecosystem as a whole. The increase in the amount of data available on mycobiomes allows for a greater knowledge to be attained on their use in increasing productivity and possible therapies. One of the major problems for health is the acquisition of resistance of microorganisms to antifungal drugs.

To contribute to possible alternatives for solving this problem, systems based on the mycobiome for monitoring and sanitization are being tested and considered good solutions, especially in the hospital environment. When the imbalance caused at a more specific level is known, it is possible to know in which human niches there are greater chances of proliferation of these pathogens. In addition, sanitizing products containing probiotics are already being developed to modulate these mycobiomes, making it difficult for pathogens to appear in these environments.

Therefore, for future work, it is crucial to start including these yeasts in studies so that we begin to have a more realistic perception of the action of fungal communities in the homeostasis of the microbiota and, consequently, of human health, and to further deepen the knowledge of clinical mycology.

## Data Availability

Data sharing not applicable.
